# Overcoming provider barriers to therapeutic drug monitoring of tumour necrosis factor inhibitors for rheumatoid arthritis: a qualitative analysis

**DOI:** 10.1093/rap/rkae030

**Published:** 2024-03-04

**Authors:** Sean P Gavan, Katherine Payne, Anne Barton

**Affiliations:** Manchester Centre for Health Economics, Division of Population Health, Health Services Research and Primary Care, School of Health Sciences, Faculty of Biology, Medicine and Health, The University of Manchester, Manchester, UK; Manchester Centre for Health Economics, Division of Population Health, Health Services Research and Primary Care, School of Health Sciences, Faculty of Biology, Medicine and Health, The University of Manchester, Manchester, UK; NIHR Manchester Biomedical Research Centre, Manchester University NHS Foundation Trust, Manchester Academic Health Science Centre, Manchester, UK; NIHR Manchester Biomedical Research Centre, Manchester University NHS Foundation Trust, Manchester Academic Health Science Centre, Manchester, UK; Versus Arthritis Centre for Genetics and Genomics, Centre for Musculoskeletal Research, Division of Musculoskeletal and Dermatological Sciences, School of Biological Sciences, Faculty of Biology, Medicine and Health, The University of Manchester, Manchester Academic Health Science Centre, Manchester, UK

**Keywords:** anti-drug antibody, drug level, qualitative, rheumatoid arthritis, therapeutic drug monitoring

## Abstract

**Objective:**

Therapeutic drug monitoring (TDM) of tumour necrosis factor-α inhibitors (TNFi), by measuring drug levels and/or anti-drug antibodies, is being considered by various international bodies to improve patient health outcomes and the value of care for people with rheumatoid arthritis. Rheumatology care providers may perceive barriers to adopting TNFi TDM within their own clinical practice, limiting the potential for patients and health care systems to benefit. This study aimed to explore the barriers perceived by rheumatologists that may reduce their uptake of TNFi TDM for rheumatoid arthritis.

**Method:**

Semi-structured one-to-one telephone interviews were performed with a convenience sample of senior rheumatologists with experience of managing people with rheumatoid arthritis. The interviews explored the rheumatologists’ understanding of TDM and their beliefs about how it can be integrated into their own routine practice. Interviews were audio recorded, transcribed verbatim and anonymized. Transcripts were coded inductively and barriers to using TNFi TDM were identified by thematic framework analysis.

**Result:**

A sample of eleven senior rheumatologists were interviewed. The rheumatologists described five barriers to adopting TNFi TDM in routine practice: (i) observing clinical need; (ii) understanding how testing can improve practice; (iii) insufficient clinical evidence; (iv) insufficient resources to pay for testing; and (v) insufficient capability to deliver testing.

**Conclusion:**

Barriers to adopting TNFi TDM in routine care settings will restrict the ability for patients to benefit from effective monitoring strategies as they begin to emerge. Strategies to overcome these barriers are suggested which will require a coordinated response from stakeholders across health care systems.

Key messages

- Effective therapeutic drug monitoring of tumour necrosis factor-α inhibitors can improve prescribing for rheumatoid arthritis.
- Rheumatologists perceive practical barriers to therapeutic drug monitoring which may reduce uptake in routine care.
- Stakeholders should implement strategies to overcome these barriers so patients benefit from therapeutic drug monitoring.

## Introduction

Therapeutic drug monitoring (TDM) of biologic tumour necrosis factor-α inhibitors (TNFi) for rheumatoid arthritis is gaining interest internationally to improve disease control and the value of care amongst rheumatology care providers and professional organizations such as the European Alliance of Associations for Rheumatology (EULAR) [1, 2]. TNFi drug levels and anti-drug antibodies (ADAb) are associated with treatment response for people with RA [3, 4]. TNFi TDM strategies comprise regular measurement of TNFi ADAb and/or drug levels to inform prescribing decisions [1]. In 2023, the EULAR taskforce on TDM found that whilst the clinical evidence supporting TDM was maturing, there was little understanding of the practical barriers faced by rheumatologists when considering whether to measure TNFi drug levels and ADAb routinely [1, 5].

Barriers to adopting new testing or monitoring strategies are a phenomenon faced by care providers across many clinical areas [6]. Complementary testing strategies, such as TNFi TDM, are not mandatory when making prescribing decisions within current regulatory or reimbursement frameworks [7]. The proposed benefits of TNFi TDM can only be realized if care providers choose to integrate and follow tests measuring TNFi ADAb and/or drug levels within their routine care settings [8]. Therefore, failing to understand and address rheumatologists’ perceived barriers to TNFi TDM will threaten the adoption of these strategies and the ability for patients to benefit. Exploring and resolving rheumatologists’ perceived barriers to TDM will help to improve health outcomes for people with RA through the uptake of effective TNFi drug level and ADAb monitoring strategies [5]. The aim of this study was to explore the barriers perceived by rheumatologists that may reduce their uptake of TNFi TDM for RA.

## Method

Semi-structured qualitative interviews were undertaken with practicing rheumatologists to explore their perceived barriers to TNFi TDM for RA. The study was reported according to the standards for reporting qualitative research [9].

### Sample

The target population was senior rheumatologists who had experience of using biologic agents to manage people with RA. A convenience sample of interviewees was recruited from the sampling frame of principal investigators belonging to the Biologics in Rheumatoid Arthritis Genetics and Genomics Study Syndicate [10]. These individuals were rheumatologists in the United Kingdom with experience of managing RA, knowledge of the pathway for prescribing TNFi treatments, and a research interest in using biomarkers to inform treatment decisions for RA. Recruitment emails were sent to all rheumatologists in the sampling frame during December 2014 and March 2015.

### Data collection

Data were collected using one-to-one telephone interviews with rheumatologists who consented to participate. The interviews were part of a wider project to understand TNFi prescribing behaviour for RA. Telephone interviews facilitated the data collection from rheumatologists distributed across the country [11]. Interviews occurred at a time most convenient for the rheumatologist and the interviewer. The rheumatologists discussed their current knowledge of TNFi TDM for RA and how they would use this strategy to inform their clinical decision-making in a routine care setting. Questions were adapted after each interview to explore whether future participants shared similar experiences [12]. Interviews were recorded digitally and transcribed verbatim. All interviews and transcriptions were undertaken by one author (SPG) who had received training in qualitative data collection.

### Data analysis

The transcripts were analysed by thematic framework analysis [13]. Descriptive codes were applied to each line of the transcripts inductively by SPG. Supplementary coding was completed independently by two other researchers (GDW and KP) [14]. Codes labelled excerpts of each transcript that described potential barriers to testing. Themes were formed by grouping codes that had a similar interpretation to characterize a pattern of responses between rheumatologists. Coding and theme formation were undertaken during data collection. A matrix was produced for each theme (row: participant; column: code) and populated with data from each transcript. Data collection continued until interviewees no longer volunteered from the sampling frame. As a result, the analysis was designed to identify an initial plausible set of barriers to adopting TNFi TDM for RA rather than achieve thematic saturation. Themes are described in the text with supporting quotations. To ensure anonymity, the rheumatologists were provided an identification number and quotations were not attributed to specific individuals.

### Ethics

Ethical approval for this study was obtained from The University of Manchester Research Ethics Committee 2 (reference number: 14147). All participants contributed voluntarily with no financial compensation and provided written informed consent for the publication of anonymized quotations.

## Results

**Figure 1.**
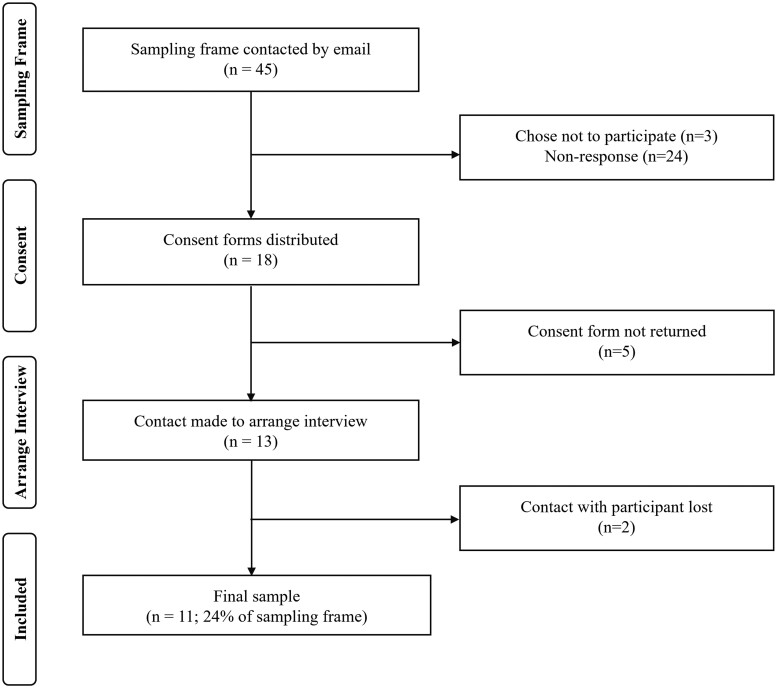
Recruitment and inclusion

Figure 1 illustrates the process to obtain the final sample. Recruitment emails were sent to 45 rheumatologists and 24% of the sampling frame (*n* = 11) consented to be interviewed. The rheumatologists were distributed evenly across the country (north: 36%; midlands: 36%; south: 27%).

### Awareness of TNFi therapeutic drug monitoring

All rheumatologists were aware of the tests to perform TNFi TDM, and some had been approached by representatives of commercial manufacturers. Only one rheumatologist described using TNFi TDM within their current practice.

*‘People are trying to sell us a little kit to check drug levels and antibody levels’* [**Rheumatologist 1]**.

*‘To be honest, we don’t…routinely measure any antibodies here. So, I mean, I know it’s a kind of interesting area…but it doesn’t really alter our clinical practice here as of yet’*. [**Rheumatologist 9]**.

*‘…we have already worked with our immunologist who have got some antibody assays to use…our plan is to change our guidelines at some point in the near future. So, we’ll include screening for antibodies’.* [**Rheumatologist 3]**.

Five barriers to adopting TNFi TDM reported by the rheumatologists are now described.

### Barrier: Observing clinical need

The rheumatologists explained that their preference for using TNFi TDM would reduce if they did not experience a clinical need in their routine practice. The clinical impact of immunogenicity to TNFi described in the literature [15] was not observed by the rheumatologists with their own patients.

*‘Put it this way, I know it’s described [immunogenicity against TNFi therapies], but we don’t see it particularly’*. [**Rheumatologist 5]**.

*‘…we’ve not really seen a lot of problems with immunogenicity’.* [**Rheumatologist 2]**.

### Barrier: Understanding how testing can improve practice

The rheumatologists varied in their understanding of how the information revealed by measuring TNFi ADAb and drug levels can improve practice to inform management decisions for patients. Some rheumatologists were optimistic about the use cases of TNFi TDM whereas others were more sceptical.

*‘One algorithm would be all patients have drug levels checked automatically at, maybe two or three times a year…whether or not they’re doing well…I think drug levels are gonna’ allow us to reduce doses of drugs as well, which is the other reason to use them’*. [**Rheumatologist 10]**.

*‘…we’ve known about immunogenicity for twenty years. So why hasn’t it, you know, taken off?’* [**Rheumatologist 8]**.

*‘It [TNFi TDM] appeals to us. We don’t know why it appeals to us. We think it’s just, you know, a shiny little gizmo…it’s interesting’*. [**Rheumatologist 1]**.

### Barrier: Insufficient clinical evidence

The rheumatologists explained how a lack of robust clinical evidence was perceived as a considerable barrier to using TNFi TDM.

*‘I think those…things like drug antibody kits and drug levels should be evaluated in proper controlled studies, really…The danger is that the market will be flooded with kits and everybody will think, “that’s great, I’ll have a go,” and…no one will know, in the end, what’s actually happening’.* [**Rheumatologist 1]**.

*‘I think that if…we have robust, you know, reliable methods…to measure antibodies and relate them to clinical response, then possibly they could go in the therapeutic algorithm. But I really think that we are a long way away’*. [**Rheumatologist 8]**.

### Barrier: Insufficient resources to pay for testing

A lack of financial resources to pay for ADAb and drug level testing was highlighted as a barrier to routine TNFi TDM. If there is uncertainty about how resources to pay for testing will be acquired, then rheumatologists will have little incentive to integrate TDM within their clinical practice.

*‘It’s [testing] a bit of a faff, and again it’s more time, more money, more thought around it…I’m still not convinced that for the majority of patients it really changes management’*. [**Rheumatologist 4]**.

### Barrier: Insufficient capability to deliver testing

Rheumatologists in the sample also highlighted that the technical capability to perform ADAb and drug level tests routinely may not be available in all health care settings. This raises a practical barrier to adopting TNFi TDM if there is variation in the facilities to perform testing between rheumatology providers.

*‘I don’t think we’ve got the capability [to perform testing]. There’s only a couple of places in the country that’ll do it, so it’s not something that we are getting too concerned about’*. [**Rheumatologist 2]**.

## Discussion

This study found that rheumatologists were generally aware of the potential use of TNFi TDM but perceived five different barriers to adopting testing within their routine clinical practice. The presence of these barriers will restrict the ability for effective TDM strategies to be used by health care providers. Patient health outcomes will, ultimately, be inhibited if barriers to TNFi TDM remain present as effective monitoring strategies emerge.

The responsibility to resolve these barriers will fall to different stakeholders across health care systems including guideline developers, care providers, assay manufacturers, service commissioners and payers. International organizations such as EULAR can help to align this activity through regular engagement with different stakeholders and proposing roadmaps for change [1]. At a national level, system readiness exercises can be undertaken to pre-empt and mitigate barriers to adopting TDM within different health care jurisdictions [16].

Possible actions to overcome the identified barriers are now described. Clinical need for TNFi TDM can be established with jurisdiction-specific data about the prevalence of ADAb and average drug levels in routine practice for disease activity subgroups [17]. Clear guidance on how TNFi TDM can inform clinical prescribing decisions (reflecting dosing or treatment-switching policies in different countries) will improve rheumatologists’ understanding about the purpose of measuring ADAb and drug levels [18]. Overcoming insufficient clinical evidence is central to the wider adoption of TDM. In 2019, the National Institute for Health and Care Excellence, who are responsible for producing recommendations for care providers in England, assessed the effectiveness and cost-effectiveness of assays for TNFi TDM [19]. To demonstrate the importance of clinical evidence, the assessment concluded that there was insufficient evidence to support the national adoption of TDM for RA and recommended that additional clinical effectiveness research should be undertaken [18]. Whilst conventional measures of effectiveness in RA (such as the Disease Activity Score-28 [20]) provide a good reflection of overall disease activity, they may fail to measure the extent of joint inflammation objectively [21]. More objective measures of joint inflammation may help to demonstrate the effectiveness of TNFi TDM within research and clinical settings. Budget holders will need to evaluate how best to release resources elsewhere to pay for routine TNFi ADAb and drug level monitoring [22]. TNFi TDM will incur additional resources upfront including assay costs, time to analyse samples and time to communicate test results with patients before receiving treatment [22]. These costs can be optimized by integrating TDM reactively at the most valuable clinical decision points. Evidence from cost-effectiveness analyses can help to establish the most valuable clinical scenarios for TNFi TDM within different health care systems and are likely to be an essential source of evidence to support wider adoption in the future [23–25]. Finally, service planners will need to determine the infrastructure requirements to scale ADAb and drug level monitoring whilst ensuring that existing testing services remain unaffected.

One limitation of this study was that data were not collected to achieve saturation. However, the goal was to explore an initial series of barriers to TNFi TDM rather than a definitive taxonomy. The findings should be interpreted as a lower-bound on the plausible barriers to TDM as perceived by rheumatologists. A second limitation was that these data were collected in 2015 which may raise concerns about whether the findings are relevant for current clinical practice. As the clinical evidence supporting TNFi TDM is starting to mature, recent trial and observational evidence has shown how measuring ADAb and/or drug levels may benefit clinically [5, 26, 27]. The availability of these empirical data may change the extent that rheumatologists perceive barriers due to insufficient clinical evidence. However, routine TNFi TDM is still not performed in the United Kingdom which reflects the clinical environment at the time of data collection. A third limitation was that the interview schedule did not distinguish between proactive TDM (regular monitoring irrespective of the clinical scenario) and reactive TDM (measuring ADAb and/or drug levels during specific clinical scenarios only) [1]. Since data collection, the EULAR task force on TDM of biopharmaceuticals for inflammatory rheumatic and musculoskeletal diseases recommended against proactive TDM [1]. The rationale for this recommendation was that an optimal blood concentration range for biologic treatment has not been defined for most indications [1]. By contrast, the EULAR task force recommended reactive TDM because of the potential to inform treatment-switching decisions following loss of response or tapering decisions for people experiencing low disease activity and remission [1]. The responses provided by the rheumatologists may have been different if this distinction was made during the interviews.

Future research could replicate this study to explore barriers to TNFi TDM with rheumatologists from other countries. A global perspective will help to support international guideline developers when proposing recommendations for implementing TDM in rheumatic conditions. Future research should also investigate the barriers to TDM perceived by rheumatologists who are less experienced with strategies to personalize health care. Finally, a robust investigation into patients’ beliefs around TDM will be essential to demonstrate acceptability and identify implementation challenges amongst those receiving care.

## Conclusion

TNFi TDM has the potential to improve health outcomes for RA. Yet rheumatologists perceive barriers to measuring drug levels and ADAb routinely which may limit the scope for TDM to become standard of care. If effective TNFi TDM strategies are being considered for adoption, early and active engagement with stakeholders across health care systems, including rheumatologists responsible for front-line care, will be vital to pre-empt and mitigate barriers to ensure that patients can benefit.

## Data Availability

The data underlying this article cannot be shared publicly because consent to make anonymized transcripts available was not sought.
